# Retraction

**DOI:** 10.1111/jcmm.16517

**Published:** 2021-05-04

Effect of CLIC1 gene silencing on proliferation, migration, invasion and apoptosis of human gallbladder cancer cells. Yue‐Ming He, Zhong‐Lin Zhang, Quan‐Yan Liu, Yu‐Sha Xiao, Lei Wei, Chen Xi and Xiang Nan. J Cell Mol Med. 2018; 22: 2569‐2579 (https://doi.org/10.1111/jcmm.13499)

The above article from Journal of Cellular and Molecular Medicine, published online on 8 March 2018 on Wiley Online Library (wileyonlinelibrary.com), has been retracted by agreement between the journal Editor‐in‐Chief and John Wiley & Sons Ltd. The retraction has been agreed following an investigation based on allegations raised by a third party. Inconsistencies in flow cytometry experiments were found and several image elements of presented western blots were found to be published previously in a different scientific context. The editors consider the conclusions invalid.

